# Readiness for influenza and COVID-19 vaccination in Germany: a comparative analysis

**DOI:** 10.3389/fpsyg.2024.1437942

**Published:** 2024-10-17

**Authors:** Anja A. Schulz, Yvonne Abt, Linus von Oppen, Markus A. Wirtz

**Affiliations:** Research Methods in Health Science, University of Education, Freiburg, Germany

**Keywords:** vaccination readiness, vaccination hesitancy, 7C-scale, motives for vaccination, beliefs about vaccination, health prevention behavior, collective responsibility

## Abstract

**Introduction:**

Vaccination readiness refers to psychological motives and beliefs that decisively determine individual and collective vaccination prevention behavior. Readiness to be vaccinated depends on expected individual and social benefits and harms. Differences exist in the perception of the threat of potential influenza vs. COVID-19 infection and its significance for the social environment. The study aimed to compare the 7C components of vaccination readiness for influenza and COVID-19 vaccination in adulthood.

**Methods:**

A total of 317 adults answered the 7C vaccination readiness scale in two vaccination-specific versions (influenza vs. COVID-19) in an online survey from September 2022 to March 2023. Data were analyzed using repeated measures, including analysis of covariance, correlations, and multiple regression.

**Results:**

For COVID-19, there is a higher readiness to be vaccinated compared to influenza regarding *complacency^R^* (*η_p_* = 0.683), *constraints^R^* (*η_p_* = 0.684), *collective responsibility* (*η_p_* = 0.782), and *compliance* (*η_p_* = 0.365). However, *confidence* (*η_p_* = 0.161) and *conspiracy^R^* (*η_p_* = 0.256) indicate an enhanced readiness for influenza vaccination (interaction scales × vaccination type: *η_p_* = 0.602). Individual influenza vaccination recommendations and age do not or only marginally moderate these effects (interaction vaccination type × recommendation: *η_p_* = 155).

**Discussion:**

The 7C subscales reveal a differentiated pattern of readiness for the two vaccination types. This emphasizes the relevance of the multidimensional structure of the construct of vaccination readiness as well as the relevance of moderating effects of the respective vaccination type on the underlying motives and beliefs. Vaccination attitudes are influenced by cultural and social conditions as well as medical standards of care. Comparing attitudes to different vaccinations in different countries thus represents an important research desideratum in order to understand the concept of vaccination readiness more comprehensively.

## 1 Introduction

Vaccinations are highly effective in preventing communicable diseases such as influenza, COVID-19, or measles and their societal spread (Wilder-Smith et al., 2017; Zheng et al., 2022). However, the compelling evidence (Du et al., 2024; Wu et al., 2024) that the benefits clearly outweigh the potential harm of vaccination has not proven to be sufficient for all people to consider vaccination positive for themselves and to decide to be vaccinated (Dubé et al., 2013; Omer et al., 2009; Petráš et al., 2022). The significance of this critical attitude in terms of vaccination hesitancy and refusal has been the subject of public debate and scientific research since the introduction of vaccinations for preventing infectious diseases, especially regarding vaccinations that are required to maintain population or herd immunity (e.g., against measles or polio) (MacDonald et al., 2020; Spier, 2001; Abate et al., 2024). Accordingly, WHO (2013) identified overcoming vaccination hesitancy as one of the 10 most essential goals to secure and improve public health.

In Germany, public health strategies for influenza and COVID-19 vaccination focus on ensuring widespread coverage, particularly for vulnerable populations (RKI, 2022). The German Standing Committee on Vaccination (STIKO) recommends annual influenza vaccinations, especially for high-risk groups such as the elderly (above 60 years), individuals with chronic illnesses, pregnant women, healthcare workers, and those who have close contact with at-risk populations. Vaccinations are generally provided free of charge through health insurance for these high-risk groups, making them easily accessible. Employers are also encouraged to offer vaccines, particularly in healthcare settings (RKI, 2023). During the COVID-19 pandemic, Germany introduced legal measures such as the Infection Protection Act, allowing for mandates like mask-wearing, lockdowns, and vaccination requirements for healthcare workers (Eurofound, 2021). While a general vaccination mandate was debated, it was not implemented nationwide. Initially, public health efforts focused on prioritizing high-risk groups and ensuring broad access through vaccination centers, mobile units, and pharmacies. In 2022, it was ensured that all those willing to be vaccinated could actually take advantage of a vaccination. This was also insistently promoted by health (political) campaigns and public media. In 2022, awareness campaigns promoted vaccination and countered misinformation, while booster shots were emphasized as new variants emerged. Despite the intensive public measures and high social importance, 22.1% of the adult population was unvaccinated at the end of 2022, mostly due to vaccine hesitancy (RKI, 2024a).

### 1.1 Generic and vaccine-specific aspects of vaccination readiness

Vaccination readiness or hesitancy can be partially understood as a generic attitude, as people tend to have an overarching attitude toward vaccination and vaccination uptake regardless of the specific vaccine (Geoghegan et al., 2020; Larson et al., 2014). However, vaccine-specific aspects of vaccination readiness must be considered due to both the individual health constitution and the properties of the specific vaccine (Anas et al., 2023; Rahbeni et al., 2024; Troiano and Nardi, 2021). According to MacDonald (2015), attitude toward a specific vaccine depends on a risk–benefit assessment, which is particularly important for newly introduced vaccines. For instance, critical concerns emerged with the newly developed COVID-19 vaccine, as it utilized mRNA technology for the first time in vaccines (Abate et al., 2024; Peretti-Watel et al., 2024).

Moreover, the type of vaccine administration, the design of the vaccination program (e.g., routine program or mass vaccination campaign), the vaccination schedule, the costs, the strength of the vaccination recommendation, the level of knowledge, and the attitude of the healthcare professionals can be decisive for the individual willingness to receive a specific vaccination (Dubé et al., 2013; MacDonald, 2015). For example, the decision to be vaccinated against influenza is primarily based on the subjective expected benefit-harm balance regarding one’s own health, in which both the protection against infection and the vaccination reactions must be considered. Risk-associated individual person characteristics (e.g., age, chronic illnesses, occupational contacts) are decisive for the decision of the respective individual, as well as for official recommendations (Grohskopf et al., 2024; de Fougerolles et al., 2024). These risk indicators contribute to the systematic variance in the population’s willingness to be vaccinated. In general, a valid assessment of readiness to be vaccinated must thus take into account (i) the objective threat of a potential infection, (ii) the individual susceptibility to an infection, and (iii) the subjective perception of the threat in an integrated manner. Accordingly, Betsch et al. (2015; see also: Eitze et al., 2024) emphasize the importance of five central vaccination readiness facets: *Confidence*, *Complacency^R^*, *Constraints^R^*, *Calculation^R^*, and *Collective Responsibility*. *Confidence* reflects trust in the effectiveness and safety of vaccinations, the healthcare system, and decision-makers’ motives. *Complacency^R^* describes the perception of health risks and threats that should be avoided through vaccinations and whether vaccinations are considered necessary (Dubé et al., 2013; Betsch et al., 2015). *Constraints^R^* refers to obstacles to taking up vaccinations (e.g., stress, [time] expenditure) and whether vaccination is considered sufficiently important to protect from health obstacles. *Calculation^R^* refers to the extent of active information search that is accompanied by a conscious risk–benefit appraisal. High *Calculation^R^* is associated with enhanced false knowledge and a lower willingness to be vaccinated (Betsch et al., 2019). *Collective Responsibility* refers to the extent of prosocial motivation to be vaccinated. Vaccinating oneself is seen as helpful as it reduces the risk of transmission of the infectious disease and can, therefore, support the health of other individuals (e.g., children, chronically ill relatives) or society (esp. herd immunity). *Confidence* in safety and effectiveness, underestimated disease risk (*Complacency^R^*), and *Constraints^R^* have proven to be important predictors of making use of influenza vaccination (Eitze et al., 2024). It must be noted that the 7C subscales *Complacency^R^*, *Constraints^R^*, *Calculation^R^*, and *Conspiracy^R^* are named contrary to the scale polarity so that their designation would misleadingly imply a negative readiness to be vaccinated. Thus, the superscript “R” indicates that the scale semantics are inverted. MacDonald (2015) considers the sub-facets of vaccination readiness to be decisive components of an individual consideration and decision-making process that results in a comprehensive vaccination attitude. According to the levels and weighting of these individual facets, people are assumed to be positioned on a continuum between the poles of “full approval” and “complete rejection” of the respective vaccinations.

### 1.2 Relevance of vaccination readiness during the COVID-19 pandemic

The COVID-19 pandemic has brought the individual and social significance of vaccinations and characteristics of public perception of vaccinations to the focus of public interest to an unprecedented extent (Prada et al., 2023). After the extensive restrictions on everyday social interaction and the comprehensive hygiene requirements to minimize the risk of infection in the early times of the pandemic, vaccinations were the most effective means of overcoming the pandemic (Wu et al., 2024). Vaccine hesitancy and readiness were key factors influencing the success of COVID-19 vaccination campaigns in all OECD countries (Abate et al., 2024). For example, Israel, which was one of the first countries to start the comprehensive vaccination of its population in December 2021, faced hesitancy primarily among ultra-Orthodox communities and younger populations, slowing the rate of booster uptake despite a strong initial rollout (Rosen et al., 2021). Misinformation and conspiracy theories circulating in rural and conservative areas contributed to lower vaccine acceptance, particularly for boosters. They were found to be important in European countries in general (Jabkowski et al., 2023) and the United States (Robinson et al., 2022). Vaccine hesitancy proved to be more pronounced, fueled by widespread misinformation (Enria et al., 2024), distrust in government policies, and lower health literacy, which posed a significant challenge to any vaccination efforts (Lee et al., 2022). Such effects were reported especially for Turkey (Alper et al., 2020) and Russia (Loseva, 2022). In China, high trust in the government and social conformity drove higher vaccine uptake, contrasting with the hesitancy rooted in misinformation and mistrust in Western countries (Huang et al., 2022; Leong et al., 2022). Japan’s initial skepticism, due to fears about side effects, was mitigated by trust in government health campaigns, though lingering concerns slowed full population coverage (Harada and Watanabe, 2021).

Thus, trust in and acceptance of the newly developed vaccines and the willingness to be vaccinated were of utmost individual and public interest, transnational and cross-cultural (Anand and Stahel, 2021; Sprengholz et al., 2021; Watson et al., 2022). Accordingly, an appropriate appraisal of disease risk (i.e., *Complacency^R^*), *Compliance,* and *Collective Responsibility* are assumed to be enhanced for COVID-19 vaccination (see below: Hypothesis 2b). The pro arguments for *Constraints^R^* and *Calculation^R^* regarding COVID-19 vaccination should also be supported (see below: Hypothesis 2c). However, there were also particular reservations about COVID-19 vaccines, most of which were associated with a generally critical or dismissive attitude toward vaccination (Geoghegan et al., 2020; Rahbeni et al., 2024). In addition, skeptics pointed out or suspected (i) that the COVID-19 vaccines were developed unusually quickly and may not have been tested according to the usual standards, (ii) that mRNA vaccines are based on a vaccine principle that has not been implemented before and uses genetic engineering to influence the genetic information in cells (Krause et al., 2023), and (iii) that infections, severe courses of disease and even infection-related deaths have also occurred in vaccinated people (Anand and Stahel, 2021; Seddig et al., 2022; Soares et al., 2021). This implies that *Confidence* is assumed to be reduced for COVID-19 vaccination (see below: Hypothesis 2a). Furthermore, contra arguments regarding *Constraints^R^* and *Calculation^R^* for COVID-19 vaccination should be conveyed (see below: Hypothesis 2c). Moreover, the immediate reaction to the vaccination could result in side effects (e.g., absence from work, Anand and Stahel, 2021; Dhamanti et al., 2023), which could have led non-risk persons to assess the immediate negative effects as disproportionately high (see Hypothesis 2b below: contra arguments regarding *Constraints^R^* and *Calculation^R^* for COVID-19 vaccination).

Furthermore, public and political discussions on a possible vaccination policy emphasized the tension between individual freedom and social responsibility (Cardona, 2021; Sprengholz et al., 2021). As a result, this health prevention issue was particularly subject to individual opinion formation, which was linked to basic political attitudes (Ward et al., 2020). Irrational or conspiratorial narratives and myths that imputed opaque and manipulative motives to the public vaccination recommendations of the pharmaceutical industry and official health authorities were abundant in vaccination skeptic circles during the pandemic (Seddig et al., 2022; Soares et al., 2021). Fake news spread on social media, and information bubbles became a prominent topic in health research. Accordingly, *Conspiracy^R^* should be reduced for COVID-19 vaccination (see below: Hypothesis 2a).

Due to the experiences within the COVID-19 pandemic, Geiger et al. (2021) supplemented the structural model of vaccination readiness (Betsch et al., 2015, 2019) by two further facets. *Compliance* corresponds to the willingness to adhere to preventive health behavioral rules and provisions. This proved to be an essential aspect of the COVID-19 pandemic to reconcile the overall societal effectiveness of vaccination protection with the individual intention to take advantage of being vaccinated (Sprengholz et al., 2021). *Conspiracy^R^* reflects individual susceptibility to belief in misinformation and conspiracy myths and proved to be a relevant predictor for vaccination hesitancy (Nicholls et al., 2021). The very good criterion validity of the 7C scale was demonstrated by the 85% multiple variance explanation in the willingness to be vaccinated (Geiger et al., 2021). In summary, readiness for vaccination against COVID-19 must be regarded in certain aspects as vaccine- or pandemic-specific.

### 1.3 Present study and research hypotheses

To shed light on this COVID-19 vaccination-specific hesitancy aspect, the study presented here focuses on whether and to what extent sub-facets of readiness for COVID-19 vaccination differ from those for influenza vaccination (see hypotheses 1 and 2 below). For these two vaccinations, different individual and social appraisals can be assumed (Dombrádi et al., 2021; SteelFisher et al., 2023). Due to the long-term development and the proven and widespread implementation over a long period of the influenza vaccination, it seems reasonable that the common vaccination reservations (i.e., general skepticism or refusal of vaccination among critical persons) are more prominent for COVID-19 than for the influenza vaccination (see below: Hypothesis 2a: *Confidence* enhanced for influenza vaccination; Hypothesis 2c: pro arguments *Constraints^R^* for influenza vaccination). The Standing Committee on Vaccination in Germany first recommended the annual influenza vaccination in 1982. Since 2002, the influenza vaccination has been recommended for people aged 60 and over, chronically ill people, and medical personnel, and since 2010, also for pregnant women (RKI, 2023). In contrast, a general vaccination recommendation applies for the COVID-19 vaccination. Thus, differences in readiness for vaccination against influenza vs. COVID-19 should be more pronounced in the group of people without influenza vaccination recommendation (see Hypothesis 3 below). Furthermore, the decision for or against influenza vaccination is usually made primarily to protect one’s own health, while the protection of the community and the protection of vulnerable groups can be considered particularly important for COVID-19 vaccination, especially for younger adults without particular health risks (see Hypothesis 2b below *Collective Responsibility* enhanced for COVID-19 vaccination).

The main research hypotheses 1–3 of the study relate to the differences in the 7C components of vaccination readiness between the two vaccination types. In addition, Hypothesis 4 concerning the differential criterion validity of the versions of the 7C scale adapted for the two vaccination types is examined, as these results are also decisive for the validity of the interpretation of the results in general.

Hypothesis 1: Vaccination readiness differs for influenza vaccination and COVID-19 vaccination (i.e., the main effect of vaccination type).

Hypothesis 2: The seven subscales of the 7C scale indicate varying differences in readiness for influenza vs. COVID-19 vaccination (i.e., interaction of the 7C single scales and vaccination type).

Hypothesis 2a: *Confidence* and *Conspiracy^R^* is higher for influenza vaccination than for COVID-19 vaccination.

Hypothesis 2b: The *Complacency^R^*, *Collective Responsibility*, and *Compliance* scales indicate a higher readiness to be vaccinated for COVID-19 than for influenza.

Hypothesis 2c: No differences for the COVID-19 vs. influenza vaccination exist for the *Constraints^R^* and the *Calculation^R^* scales, as both pros and cons regarding immediate and long-term effects, benefits, and harms may be relevant.

Hypothesis 3: The difference in readiness for influenza vs. COVID-19 vaccination is enhanced if there is no influenza vaccination recommendation (i.e., the interaction of influenza vaccination recommendation with the 7C differences in readiness for influenza vs. COVID-19 vaccination).

Hypothesis 4a: The 7Cs of influenza vaccination readiness correlate more strongly with influenza vaccination-related importance rating and influenza vaccination status than the 7Cs of COVID-19 vaccination readiness.

Hypothesis 4b: The 7Cs of COVID-19 vaccination readiness correlate more strongly with COVID-19 vaccination-related importance assessment and COVID-19 vaccination status than the 7Cs of influenza vaccination readiness.

Hypothesis 4c: The 7Cs of COVID-19 vaccination readiness correlate more strongly with the relevance and implementation of COVID-19 infection prevention measures than the 7Cs of influenza vaccination readiness.

Thus, the central objective of this study is to empirically determine the differences in the seven sub-facets of vaccination readiness regarding COVID-19 and influenza vaccination. The insights into these differences and their correlations with further vaccine-related characteristics should enhance the understanding of the construct of vaccination readiness and its vaccine-specific characteristics.

## 2 Methods

This study is an extension of a study conducted in the DFG project “Structural Modeling and Assessment of Health Literacy in Allergy Prevention of New Parents” [G.Z.: WI-3210/7-1]. For this DFG-funded study, an Ethics Statement was approved by the Ethics Committee of the German Psychological Society,^1^ and the study protocol has been preregistered at the Leibniz Institute of Psychology (ZPID; Wirtz et al., 2021). There, (expectant) parents of infants were surveyed, in particular on attitudes toward COVID-19 vaccination. In the present study, in which adults were surveyed, the same standards were adhered to and implemented. Informed consent was obtained from all participants. The study was performed in accordance with the ethical principles in the Declaration of Helsinki.

### 2.1 Measures

The 7C scale (Geiger et al., 2021; Rees et al., 2022) was used in particular within the COVID-19 Snapshot Monitoring Study (COSMO study) to assess and monitor vaccination-related barriers and support factors using subjective self-reported data. The 7C scale uses three items each to measure the 7C facets: *Confidence*, *Complacency^R^*, *Constraints^R^*, *Calculation^R^*, *Collective Responsibility*, *Compliance,* and *Conspiracy^R^.* It should be noted that the 7C subscales *Complacency^R^*, *Constraints^R^*, *Calculation^R^*, and *Conspiracy^R^* are named contrary to the scale polarity, so that their designation would misleadingly imply a negative readiness to be vaccinated. To ensure that this reversed naming is taken into account and to avoid apparently contradictory statements, these scales are labeled with R (reversed) (e.g., “high *Conspiracy^R^*” corresponds to “low conspiracy”). The 21 individual items are answered on a 7-point rating scale (1 = strongly disagree to 7 = strongly agree). The scale structure can be modeled using a bifactor model with a dominant general factor, “Readiness to vaccinate” (Geiger et al., 2021). While the general factor, according to McDonald’s *ω* (omega), exhibits good internal consistency at 0.88, the internal consistency for the individual factors is considerably weaker: range *ω* = 0.27 (*Constraints^R^*) to *ω* = 0.59 (*Compliance*).

In the study presented here, the 21 7C items were used in two versions, which were adapted for COVID-19 and influenza vaccination by replacing the general wording “vaccination” with “influenza vaccination” or “COVID-19 vaccination,” respectively. A content revision was only necessary for two items of the *Compliance* scale: The adapted influenza formulations refer to the special attention and compliance with hygiene measures (item 18: originally: excluding people from public events) and behavioral rules (item 19: originally: legal sanctions). All items proved to be comprehensible in both a cognitive pre-test and a cross-sectional pre-test of the instrument involving 187 participants. Both adapted versions and the original item formulation are attached in Supplementary material 1. In addition, participants self-reported the current COVID-19 vaccination status (“1”—“no”; “2”—“yes, but not completely”; “3”—“yes, completely”), influenza vaccination use (“1”—“never”; “2”—“at least once”) and general vaccination status (e. g. measles, polio; “1”—“no”; “2”—“yes”), the subjective importance of being vaccinated (“1”—“extremely unimportant” to “6”—“extremely important”) and the relevance of adhering to COVID-19 protective measures (“1”—“not important at all” to “6”—“very important”) and their implementation in daily life (“1”—“very bad” to “6”—“very good”) were surveyed using single items. Furthermore, sociodemographic information, socioeconomic status (MacArthur Scale; Hoebel et al., 2015), and indicators of influenza vaccination recommendation (age at least 60 years, regular contact with people at risk, working in a healthcare profession, previous illness) were assessed.

### 2.2 Data collection and study sample

The cross-sectional data were collected online from August 2022 to March 2023 via the “SoSciSurvey” platform. The average processing time was 16.5 min (SD = 4.9 min). Missing data were avoided due to the default settings. While limited statutory COVID-19 protective measures were still mandatory in autumn 2023 (e.g., obligatory mask-wearing on local public transport), the special statutory requirements for public COVID-19 infection protection ended in the first months of 2023. We opted for an online-based convenience sample, as the extensive restrictions on public life considerably limited the possibilities for ensuring a population-based, representative sample. Instead, we targeted specific groups of people to cover a broad age spectrum and to be able to reach target groups of younger adults for whom a recommendation for influenza vaccination prevailed. The study call was distributed via social networks and Kiel University mailing lists. People who were at least 18 years old and had sufficient knowledge of the German language were eligible to take part in the survey. Since the digital study call primarily reached young adults, older individuals and those with increased health risks were also contacted.

The study sample predominantly consisted of people of young adults (*M* = 29.95 and a median of 23 years; see Supplementary material 2). The proportion of people with influenza vaccination recommendations was 42.6%. 54.9% of those had already been vaccinated at least once against influenza. Since the proportion of vaccinated people in Germany is considerably below 50% even among groups with influenza vaccination recommendations (2021/22: e.g., over 60-year-old: 43.3%, chronic illness: 35.4%, pregnant women: 17.5%; Statista, 2022a), this indicates increased vaccination acceptance in the study sample. This is also reflected as 90.3% of participants had already received a COVID-19 vaccination (full vaccination: 84.9%). In the general population, the proportion of people with a first COVID-19 vaccination was stable between 77 and 78% in the second half of the year 2022 (Statista, 2022b). According to the MacArthur scale, the more educated (90.6% high school graduates) and female (73.8% women) sample has an above-average socioeconomic status according to the arithmetic mean *M* = 6.22 (general population: *M* = 5.25; Hoebel et al., 2015). However, the median of 5 indicates that the proportion of people with lower socioeconomic status can be considered appropriate.

### 2.3 Statistical analysis

Multivariate analysis of variance (ANOVA) with repeated measures is used to test the study hypotheses (Tabachnick and Fidell, 2019). The vaccination type (influenza vs. COVID-19) is a repeated measures factor with two levels. The influenza vaccination recommendation (yes, no) is included as a 2-level between-subjects factor. In order to test the interaction of the items with the vaccination type (Hypothesis 2), the seven factors are defined as a further 7-level repeated measures factor. The variance analysis approach thus enables an integrated and maximally test-economic examination of the effects that refer to mean differences. In addition to the main effects, which reflect fundamental differences in the factor levels under consideration, the interaction effects are particularly important in this study: These indicate in particular which internal sub-facets of the readiness to vaccinate are specifically pronounced for the respective vaccination types. Due to the multi-factorial variance decomposition within the framework of the ANOVA approach, this information is tested much more rigorously than if non-variance analysis methods were used (Tabachnick and Fidell, 2019). In addition, the Pearson product–moment correlation is determined for the within components. This depicts the similarities between the individual variables: The more positive the correlation, the higher the common information share of the corresponding characteristics, which may indicate an overarching underlying latent source of information (Roettele and Wirtz, 2020).

The ANOVA assumption of equal covariance matrices between groups with and without an influenza vaccination recommendation was tested using the BOX-M test (Tabachnick and Fidell, 2019). The Levene test is used to test the equality of the variances between the groups. In principle, the ANOVA is (a) robust against corresponding violations for sufficiently large samples (*n_i_* per group >30) and (b) for similarly large samples (Tabachnick and Fidell, 2019). In the present study, the ratio of group sizes (135 vs. 180) is 0.75, so the validity of the test can be considered at most weakly affected. Nevertheless, the results of the BOX-M- and the Levene-test are documented in order to ensure the transparency of the data characteristics.

The internal consistencies of the 7C scale and the seven individual scales are calculated using McDonald’s *ω* (omega), which is determined using the item-specific maximum likelihood estimated unstandardized factor loadings and error variances (assumption: *τ*-congeneric measurements; Geiger et al., 2021). To ensure an overall nominal *α*-level of 0.05 despite multiple testing, an *α*-error adjustment according to Bonferroni (*α*/*k*; where *k* = number of individual tests performed; Tabachnick and Fidell, 2019) is adopted for each hypothesis or each statistical procedure, respectively. Hedges g is used as a measure of the effect size for pairwise mean differences, with *g* = 0.2, 0.5, and 0.8 as orientation values for small, medium, and large effect sizes (Tabachnick and Fidell, 2019). For the comparison of *k* > two mean values, the effect size is determined using the partial *η*_p_, with *η_p_* = 0.10, 0.24, and 0.37 as reference values for small, medium, or large effect sizes. Linear correlations are calculated using product–moment correlation (reference values: 0.1 = small, 0.3 = medium, 0.5 = large). All statistical analyses are computed using the SPSS 29.0 software. Differences in dependent correlations were tested by Hotellings-T statistic (Wilcox and Tian, 2008) using Hemmerich’s (2017) online calculation tool.

## 3 Results

### 3.1 Differences in the 7Cs of vaccination readiness for influenza vs. COVID-19 vaccination

The 7C total scale indicates a higher readiness for COVID-19 vaccination than for influenza vaccination (*F*_1,315_ = 179.659^***^; *η_p_* = 0.602; Table 1) (Hypothesis 1). However, this effect is strongly moderated by the seven individual scales, as the seven scales reflect significantly varying differences in readiness to be vaccinated between the influenza vs. COVID-19 vaccination types. In accordance with Hypothesis 2a, *Confidence* and *Conspiracy^R^* proved to be enhanced for influenza vaccination with medium effects size (*η_p_* = 0.161, 0.256). *Complacency^R^*, *Collective Responsibility,* and *Compliance* indicate a higher readiness to vaccinate against COVID-19 with a high effect size (*η_p_* = 0.683, 0.728, 0.365), which confirms Hypothesis 2b. The higher values of *Constraints^R^* (*η_p_* = 0.684) for the COVID-19 vaccination and the higher values of *Calculation^R^* (*η_p_* = 0.228) for the influenza vaccination do not confirm Hypothesis 2c, according to which the pros and cons should be balanced. In an additional overall 2 × 2 × 7-ANOVA, in which the seven scales are also included as a repeated measurement factor, the scale dependence of the influenza vs. COVID-19 differences reveals a high effect size *η_p_* = 0.602 (Table 2, interaction of vaccination type and scales; *F_6,1890_* = 179.818^***^) (Hypothesis 2).

**Table 1:** Descriptive statistics and results of the 2 × 2-ANOVAs testing the effects of vaccination type (influenza vs. COVID-19) and influenza vaccination recommendation (yes vs. no) for each 7C-scale and total score.

			Influenza			COVID-19						2 × 2-ANOVA		
												ME IC	ME IR	IE IC IR
		*N*	*M*	SD	Levene^a^	*M*	SD	Levene	Box-M^b^	*g*^c^	*r*	*F*_df = 1, 315_ *η*_p_^d^	*F*_df = 1, 315_ *η*_p_	*F*_df = 1, 315_ *η*_p_
Confidence	No IR	135	4.94	1.37	0.355	4.74	1.52	0.741	0.446	0.172		8.334^**, †^	0.470^n.s.^	0.000^n.s.^
	IR	182	4.83	1.48		4.64	1.52			0.154		0.161	0.001	0.000
	All	317	4.89	1.42		4.70	1.52			0.164	0.680			
Complacency^R^	No IR	135	3.59	1.41	<0.001	5.15	1.60	0.041	0.005	−1.090		274.917^***^	14.283^***^	6.990^**, †^
	IR	182	4.40	1.74		5.52	1.42			−0.784		0.683	0.207	0.148
	All	317	3.94	1.60		5.31	1.53			−0.953	0.582			
Constraints^R^	No IR	135	3.11	1.26	<0.001	4.86	1.47	0.599	0.026	−1.196		278.421^**^	8.346^**, †^	12.770^***^
	IR	182	3.82	1.58		4.95	1.45			−0.703		0.684	0.161	0.197
	All	317	3.41	1.45		4.90	1.46			−0.958	0.434			
Calculation^R^	No IR	135	4.03	1.32	0.356	3.61	1.34	0.455	0.251	0.274		17.147^***^	0.137^n.s.^	0.693^n.s.^
	IR	182	3.91	1.44		3.63	1.47			0.194		0.228	0.002	0.045
	All	317	3.97	1.37		3.62	1.39			0.242	0.425			
Collective resp.	No IR	135	3.41	1.59	<0.001	5.49	1.75	0.479	0.044	−1.347		356.038^***^	2.882^n.s.^	16.114^***^
	IR	182	4.08	1.93		5.43	1.87			−0.799		0.728	0.095	0.221
	All	317	3.70	1.75		5.47	1.80			−1.077	0.574			
Compliance	No IR	135	3.26	1.37	0.302	3.82	1.64	0.363	0.572	−0.458		48.317^***^	0.551^n.s.^	1.861^n.s^
	IR	182	3.48	1.46		3.85	1.76			−0.328		0.365	0.045	0.077
	All	317	3.35	1.41		3.83	1.69			−0.405	0.726			
Conspiracy^R^	No IR	135	5.96	1.27	0.715	5.70	1.59	0.912	0.022	0.315		21.817^***^	0.533^n.s.^	0.315^n.s^
	IR	182	5.81	1.35		5.61	1.52			0.216		0.256	0.047	0.032
	All	317	5.90	1.30		5.66	1.56			0.270	0.836			
**7C-scale**	No IR	**135**	**4.05**	**0.86**	**<0.001**	**4.77**	**1.21**	**0.720**	**0.077**	**−0.932**		179.659^***^	**1.936**^**n.s.**^	7.887^**, †^
	IR	182	**4.34**	**1.06**		**4.80**	**1.23**			**−0.586**		**0.602**	**0.077**	**0.156**
	All	**317**	**4.17**	**0.96**		**4.78**	**1.21**			**−0.775**	**0.762**			

^***^p < 0.001, ^**^p < 0.01, n.s., not significant; ^†^Not significant after Bonferroni correction *α*_cor_ = *α*/24 = 0.002. ^R^Reversed scale with high values indicating high willingness to vaccinate; ME IC, main effect influenza- vs. COVID-19-vaccination; ME IR, Main effect influenza vaccination recommendation; IE, interaction effect; ^a^*p*-value of Levene-test of homogeneity of variances; ^b^*p*-value of BOX-M-test of homogeneity of covariance matrices; ^c^Hedges *g* (|*g*|: 0.2 = small, 0.5 = medium, 0.8 = large effect size); ^d^partial-*η* (eta) (*η_p_* = 0.01 = small, 0.24 = medium, 0.37 = large effect size). Bold faced: 7C toatal score statistics.

**Table 2:** Results of the 2 × 2 × 7-ANOVAs testing the within-subject effects of vaccination type (VT: influenza vs. COVID-19) and the seven scales of the 7C scale as well as the between-subject effect of influenza vaccination recommendation (IVR: yes vs. no).

	*F*	df	*p* ^a^	*η*_p_ ^b^
Main effects				
Vaccination type	175.575	1, 315	<0.001	0.597
7C-scales	193.977	6, 1.890	<0.001	0.593
IVR	1.989	1, 315	0.159	0.077
Two-way interaction effects				
VT × 7C-scales	179.818	6, 1.890	<0.001	0.602
VT × IVR	7.669	1, 315	0.006	0.155
7C-scales × IVR	6.121	6, 1.890	<,001	0.138
Three-way interaction effect				
VT × 7CS × IVR	6.741	6, 1.890	<0.001	0.145

a Bonferroni-adjusted *α*_cor_ = 0.007.

b Partial-*η* (eta) (*η*_p_ = 0.01 = small, 0.24 = medium, 0.37 = large effect size).

These effects are mostly independent of whether an influenza vaccination recommendation exists (Hypothesis 3). After Bonferroni correction, only *Constraints^R^* (*η_p_* = 0.197) and *Collective Responsibility* (*η_p_* = 0.221) exhibit a significant interaction effect: For both scales, the difference between influenza and COVID-19 vaccination is, as expected in Hypothesis 3, larger for people without influenza vaccination recommendation (*g* = −1.196, −1.347) than for people with recommendation (*g* = −0.703, −0.799). A main effect of the influenza vaccination recommendation is only present for *Complacency^R^* (after Bonferroni correction): *Complacency^R^* is higher for both the influenza vaccination (*M* = 4.4 vs. 3.59) and the COVID-19 vaccination (*M* = 5.52 vs. 5.15) when an influenza vaccination recommendation exists.

### 3.2 Correlations of the 7 Cs regarding influenza and COVID-19 vaccination

Table 3 displays the correlations of the seven single scales and total scale scores for the two vaccination types. The correlation between the 7C influenza and COVID-19 vaccination readiness total score is high at *r* = 0.764. For the single scales, the highest correlation of *r* = 0.836 is found for *Conspiracy^R^* between vaccination types. *Confidence* and *Compliance* are associated between the two vaccination types about *r* = 0.7, *Complacency^R^* and *Collective Responsibility* about *r* = 0.58. For *Contraints^R^ and Calculation^R^*, a medium correlation between the vaccination types results (*r* about 0.43).

**Table 3:** Upper table: intercorrelations between the influenza and COVID-19 7Cs and internal scale consistency according to McDonald’s *ω* (omega); lower table: intercorrelations within the COVID-19 and the influenza 7Cs.

			COVID-19							
			Confidence	Complacency^R^	Constraints^R^	Calculation^R^	Coll. resp.^R^	Compliance	Conspiracy^R^	7C scale
			*ω* = 0.771	*ω* = 0.834	*ω* = 0.817	*ω* = 0.537	*ω* = 0.911	*ω* = 0.770	*ω* = 0.838	*ω* = 0.890
Influenza	Confidence	*ω* = 0.800	**0.680**	0.603	0.637	(−0.110)	0.662	0.557	0.684	0.696
	Complacency^R^	*ω* = 0.850	0.294	**0.582**	0.451	[0.185]	0.395	0.334	0.401	0.488
	Constraints^R^	*ω* = 0.807	0.284	0.402	**0.434**	(0.103)	0.296	0.277	0.299	0.386
	Calculation^R^	*ω* = 0.583	−0.223	−0.200	−0.262	**0.425**	−0.232	[−0.165]	−0.241	[−0.177]
	Collective resp.	*ω* = 0.929	0.449	0.528	0.575	(0.097)	**0.574**	0.442	0.459	0.582
	Compliance	*ω* = 0.851	0.538	0.608	0.607	(0.079)	0.630	**0.726**	0.553	0.701
	Conspiracy^R^	*ω* = 0.891	0.650	0.678	0.604	(−0.014)	0.646	0.535	**0.836**	0.735
	7C scale	*ω* = 0.924	0.596	0.715	0.687	[0.161]	0.675	0.611	0.665	**0.764**

COVID-19 7Cs (upper right off-diagonal) and the influenza 7Cs (lower left off-diagonal)								
	Confidence	Complacency^R^	Constraints^R^	Calculation^R^	Coll. resp.	Compliance	Conspiracy^R^	7C scale
Confidence	–	0.649^#^	0.681^#^	(−0.029)^#^	0.767^#^	0.651	0.792	0.843_#_
Complacency^R^	0.318^#^	–	0.771	(0.053)	0.724	0.668^#^	0.735^#^	0.856^#^
Constraints^R^	0.336^#^	0.662	–	(0.030)	0.771^#^	0.714^#^	0.696^#^	0.868^#^
Calculation^R^	−0.269^#^	(0.017)	(0.005)	–	(−0.018)	(0.105)	(0.007)	[−0.191]
Collective resp.	0.498^#^	0.592	0.609^#^	[−0.131]	–	0.731	0.774^#^	0.894^#^
Compliance	0.520	0.467^#^	0.386^#^	[−0.170]	0.645	–	0.478^#^	0.701
Conspiracy^R^	0.710	0.395^#^	0.312^#^	[−0.154]	0.400^#^	0.646^#^	–	0.868^#^
7C scale	0.687^#^	0.768^#^	0.731^#^	(0.031)	0.825^#^	0.759	0.682^#^	–

Round bracket “()” indicates non-significant correlations at the significance level *α* = 0.05; square brackets; “[]” indicate non-significant correlations after Bonferroni correction at the significance level *α*_cor_ = 0.05/120 = 0.0004; ^#^Significant difference between the correlation of the COVID-19 vs. the correlation of the corresponding influenza scale after Bonferroni-correction *α*_cor_ = 0.05/28 = 0.0018. Bold faced: Correlation between COVID-19 7Cs and influenza 7Cs.

With regard to the scale intercorrelations between the vaccination types (upper Table 4), only the *Calculation^R^* scale is revealed to be not, or at most, weakly correlated (max |*r*| = 0.262) with the other 7C subscales between the vaccination types. Contrary to expectations, the influenza vaccination-related *Calculation^R^* is even consistently negatively correlated with the other COVID-19 vaccination-related 7C subscales. All other scale intercorrelations are significantly positive between the vaccination types, consistent with expectations. Influenza vaccination-related *Confidence* (*r* = 0.557–0.684; row data), *Compliance* (*r* = 0.538–0.630) and *Conspiracy^R^* (*r* = 0.535–0.678) correlate most highly with aspects of COVID-19 vaccination readiness. For COVID-19 vaccination-related *Confidence*, *Complacency^R^*, and *Conspiracy^R^* (column data), correlations prove to be mostly considerably lower, especially for influenza vaccine-related *Complacency^R^* and *Constraints^R^* (*r* = 0.284–0.402).

**Table 4:** Internal consistencies and correlations of 7Cs on readiness for influenza (INF) and COVID-19 vaccination (COV) with sociodemographic characteristics and aspects of infection prevention, testing of correlation differences between vaccination types using Hoteling’s t-statistics (*t*(Δ(*r*))) and multiple regression prediction.

			Confidence		Complacency^R^		Constraints^R^		Calculation^R^		Collect. Resp.		Compliance		Conspiracy^R^		7C-Scale	
			INF	COV	INF	COV	INF	COV	INF	COV	INF	COV	INF	COV	INF	COV	INF	COV
McDonald’s *ω*			0.800	0.771	0.850	0.834	0.807	0.817	0.583	0.537	0.929	0.911	0.851	0.770	0.891	0.838	0.924	0.890
Sociodemographic characteristics																		
Gender	*r*_df = 315_		0.035	0.080	0.057	0.019	0.096	0.057	0.072	−0.048	0.033	−0.043	0.005	0.051	0.038	0.036	0.069	0.027
	*t*(Δ(*r*))_df = 314_		−1.00		0.74		0.65		2.00^*, ‡^		1.46		−1.10		0.06		1.08	
Age	*r*_df = 315_		−0.118^*, †^	−0.015	0.169^**^	0.049	0.252^***^	0.012	0.063	−0.028	0.039	−0.107	0.036	0.034	−0.120^*, †^	−0.051	,074	−0.022
	*t*(Δ(*r*))_df = 314_		−2.22^*, ‡^		2.36^*, ‡^		4.88^***^		1.51		2.84^**^		0.12		−2.16^*, ‡^		2.49^*, ‡^	
School	*r*_df = 315_		0.207^***^	0.183^**^	−0.044	0.116^*, †^	0.024	0.142^*, †^	−0.058	−0.169^**^	0.100	0.184^**^	0.053	0.092	0.170^**^	0.115^*, †^	0.099	0.128^*, †^
Education	*t*(Δ(*r*))_df = 314_		0.55		3.15^**^		4.17^***^		1.86		−1.64		−0.94		1.73		−0.75	
SES	*r*_df = 315_		0.156^**, †^	0.192^***^	0.199^***^	0.166^***^	0.196^***^	0.238^***^	0.061	−0.033	0.085	0.146^**, †^	0.061	0.213^***^	0.155^**, †^	0.163^**, †^	0.191^***^	0.202^***^
	*t*(Δ(*r*))_df = 314_		−0.82		0.67		−0.72		1.56		−1.18		−3.76^***^		−0.25		−0.29	
Influenza vaccination																		
Importance	*r*_df = 315_		0.537^***^	0.504^***^	0.697^***^	0.625^***^	0.686^***^	0.618^***^	−0.146^**, †^	0.068	0.762^***^	0.552^***^	0.617^***^	0.525^***^	0.487^***^	0.539^***^	0.819^***^	0.639^***^
	*t*(Δ(*r*))_df = 314_		−0.08		2.03^*, ‡^		1.78		−3.61^***^		6.38^***^		2.83^**^		−1.92		6.81^***^	
	*β*_i_^a^		0.118^*, ‡^		0.248^***^		0.232^***^		−0.051		0.316^***^		0.117^**^		0.043			
	*R*^2^_corr_ = 0.758																	
Vaccinated	*r*_df = 315_		0.268^***^	0.223^***^	0.548^***^	0.300^***^	0.489^***^	0.292^***^	−0.053	0.055	0.480^***^	0.231^***^	0.322^***^	0.284^***^	0.214^***^	0.248^***^	0.508^***^	0.303^***^
	*t*(Δ(*r*))_df = 314_		1.04		5.75^***^		3.78^***^		−1.79		5.46^***^		0.96		−1.09		6.21^***^	
	*b*_i_^b^		0.238		0.640^***^		0.316		−0.026		0.276		−0.036		−0.263			
	Nagelkerke-*R*^2^ = 0.457; regression constant *b*_0_ = 4.228^***^																	
COVID-19 vaccination																		
Importance	*r*_df = 315_		0.676^***^	0.749^***^	0.441^***^	0.825^***^	0.380^***^	0.830^***^	−0.263^***^	0.062	0.564^***^	0.833^***^	0.608^***^	0.728^***^	0.701^***^	0.810^***^	0.696^***^	0.903^***^
	*t*(Δ(*r*))_df = 314_		−2.60^*, ‡^		−13.22^***^		−13.45^***^		−5.60^***^		−7.46^***^		−4.21^***^		−5.77^***^		−12.43^***^	
	*β*_i_^a^			0.049		0.251^***^		0.249^***^		0.041		0.232^***^		0.044		0.207^***^		
	*R*^2^_corr_ = 0.841																	
Vaccinated	*r*_df = 315_		0.551^***^	0.662^***^	0.316^***^	0.673^***^	0.240^***^	0.628^***^	−0.197^***^	−0.098	0.409^***^	0.740^***^	0.498^***^	0.576^***^	0.650^***^	0.744^***^	0.551^***^	0.737^***^
	*t*(Δ(*r*))_df = 314_		−3.34^***^		−16.06^***^		−8.31^***^		−1.67		−9.45^***^		−2.31^*, ‡^		−4.37^***^		−7.10^***^	
	*b*_i_^b^			0.038		0.190		0.656^*, ‡^		−0.143		0.146		0.167		0.658^*, ‡^		
	Nagelkerke-*R*^2^ = 0.695; regression constant *b*_0_ = 5.738^***^																	
COVID-19 protective measures																		
Importance	*r*_df = 315_		0.575^***^	0.572^***^	0.391^***^	0.653^***^	0.349^***^	0.618^***^	−0.237^***^	−0.068	0.507^***^	0.661^***^	0.534^***^	0.566^***^	0.585^***^	0.619^***^	0.554^***^	0.569^***^
	*t*(Δ(*r*))_df = 314_		0.09		−6.71^***^		−5.74^***^		−2.87^**^		−3.84^***^		−0.95		−1.35		−0.48	
	*β*_i_^a^			0.003		0.283^***^		0.071		−0.089		0.253^**^		0.073		0.117		
	*R*^2^_corr_ = 0.507																	
Implementation	*r*_df = 315_		0.510^***^	0.518^***^	0.304^***^	0.565^***^	0.290^***^	0.540^***^	−0.286^***^	−0.031	0.398^***^	0.583^***^	0.426^***^	0.457^***^	0.609^***^	0.679^***^	0.496^***^	0.599^***^
	*t*(Δ(*r*))_df = 314_		−0.21		−5.79^***^		−4.96^***^		−4.39^***^		−4.40^***^		−0.84		−2.89^**^		−3.33^***^	
	*β*_i_^a^			0.027		0.211^**, ‡^		0.085		−0.036		0.241^**, ‡^		−0.051		0.181^*, ‡^		
	*R*^2^_corr_ = 0.387																	

*p < 0.05; **<0.01; ***<0.001; INF, Influenza vaccination; COV, COVID-19 vaccination; ^†^Not significant after Bonferroni-correction for 16 tested individual correlations per characteristic: *α*_cor_ = 0.05/16 = 0.003; ^‡^Not significant after Bonferroni-correction for 8 tested correlation differences per characteristic: *α*_cor_ = 0.05/8 = 0.006; ^a^standardized multiple linear regression weights; ^b^unstandardized multiple logistic regression weights.

At the level of the 7C overall scale, the influenza vaccination-related 7C scale is similarly strongly associated with the other COVID-19 vaccination-related individual scales—except *Calculation^R^*—in the range *r* = 0.596–0.715 (last row upper Table 3). For the COVID-19 vaccine-related overall scale, however, there is a considerably weaker association with the influenza vaccine-related *Complacency^R^* and *Constraints^R^* (*r* = 0.488, 0.386) (last column, upper Table 3).

Except for the *Calculation* scale, in general, the 7C individual scales for COVID-19 vaccination readiness proved to be considerably more highly correlated with each other (see upper-right-off-diagonal in lower Table 3: range: 0.478–0.792; median: 0.724) than the 7C individual scales for influenza vaccination readiness (lower-left-off-diagonal in lower Table 3: range: 0.312–0.710; median: 0.498).

### 3.3 Correlations of 7Cs for influenza and COVID-19 vaccination with sociodemographic characteristics and aspects of infection prevention

Among the socio-demographic characteristics, the highest correlations were found for SES (Table 4): Readiness to be vaccinated correlates positively with SES for the 7C total scale for both vaccination types (influenza: *r* = 0.191; COVID-19: *r* = 0.202). *Complacency^R^* and *Constraints^R^* are also associated with higher SES for both vaccine types (influenza: *r* = 0.199/0.196; COVID-19: *r* = 0.166/0.238). COVID-19-related *Confidence* (*r* = 0.192) and *Compliance* (*r* = 0.213) are also positively associated with SES, with the correlation for *Compliance* also being stronger in contrast (*t*_*df* = 314_ = −3.76, *p* < 0.001) than the correlation for influenza vaccination-related *Compliance* (*r* = 0.061). School education correlates significantly higher with *Complacency^R^* (*r* = 0.116 vs. −0.044; *t*_*df* = 314_ = 3.15, *p* = 0.002) and *Constraints^R^* (*r* = 0.142 vs. 0.024; *t*_*df* = 314_ = 4.17, *p* < 0.001) for COVID vaccination than for influenza vaccination. Furthermore, the influenza vaccine-related *Constraints^R^* are significantly more highly correlated with age (*r* = 0.252 vs. 0.012; *t*_*df* = 314_ = 4.88, *p* < 0.001).

For the validation criteria addressed in hypotheses 4a–c, considerably higher correlations were found overall (Table 4). Only the correlations for *Calculation^R^* proved to be strikingly weak here. The correlation differences between the two vaccination types of the other subscales and the 7C scale are almost always in the direction formulated in hypotheses 4a to c. Regarding Hypothesis 4c, the importance of the COVID-19 vaccination was correlated more positively with COVID-19 vaccination-related 7C facets than with the corresponding influenza vaccination-related 7C scale values (*t_df = 314_* = −2.60 to −13.45). Similar effects resulted for COVID-19 vaccination status, though there is no significant difference in correlation for *Calculation^R^* (*t_df = 314_* = −1.67). Complementarily, the importance of the influenza vaccination correlated significantly more strongly with the influenza vaccination-related 7C facets *Collective Responsibility* and *Compliance* as well as the 7C total score than with the corresponding COVID-19 vaccination-related 7C scale values (*t_df = 314_* = 2.83–6.81) (Hypothesis 4a). For the use of the influenza vaccination, there are, as expected, higher correlations with the influenza vaccination-related 7Cs *Complacency^R^*, *Constraints^R^*, *Collective Responsibility,* and the 7C total score than with the corresponding COVID-19 vaccination-related 7Cs (*t_df = 314_* = 3.78–6.21). Contrary to Hypothesis 4a, the influenza vaccination-related *Calculation^R^* (*r* = −0.146) is associated significantly lower than the COVID-19 vaccination-related *Calculation^R^* (*r* = 0.068) with the importance of the influenza vaccination (*t_df = 314_* = −3.61).

With regard to Hypothesis 4c, both the importance and the implementation of COVID-19 protective measures show, as expected, mostly high correlations with the COVID-19 vaccination-related 7C scales. COVID-19 vaccine-related *Complacency^R^* (*t*_*df* = 314_ = −6.71, −5.79), *Constraints^R^* (*t*_*df* = 314_ = −5.74, −4.96), *Calculation^R^* (*t*_*df* = 314_ = −2.87, −4.39), *Collective Responsibility* (*t*_*df* = 314_ = −3.84, −4.40) are more positively correlated with both criteria variables than corresponding influenza vaccine-related 7C scales. Here, as for the other criterion variables, *Calculation^R^* is the only scale for which negative correlations were found.

According to Nagelkerke’s *R*^2^, the dichotomous information of influenza vaccination uptake is explained at 0.457 by the influenza vaccination-related 7C scales (significant single predictor: *Complacency^R^*) and the information of COVID-19 vaccination status at 0.695 by the COVID-19 vaccination-related 7C scales. The corrected multiple variance explanation of the importance assessment of influenza vaccination by the influenza vaccination-related 7C scales is 75.8% (significant predictors: *Complacency^R^*, *Constraints^R^*, *Collective Responsibility* and *Compliance*), and the importance of COVID-19 vaccination by the COVID-19 vaccination-related 7C scales is 84.1% (significant individual predictors: *Complacency^R^*, *Constraints^R^*, *Collective Responsibility* and *Conspiracy^R^*). The importance (significant individual predictors: *Complacency^R^*, *Collective Responsibility*) of the general COVID-19 measures and their implementation (no significant individual predictors after Bonferroni corrector) is also high at 50.7 and 38.7%, respectively.

## 4 Discussion

Using the 7C scale adapted for both influenza and COVID-19 vaccination, a differentiated picture of the facets of vaccination readiness in the final phase of the COVID-19 pandemic could be obtained. The 7C total score indicates a significantly increased readiness to be vaccinated for the COVID-19 vaccination than for the influenza vaccination (Hypothesis 1 confirmed). However, the 7C individual facets should be considered essential, as evidenced by their strong interaction with vaccination types (Hypothesis 2 confirmed): Higher *Confidence* and *Conspiracy^R^* for influenza vaccination (Hypothesis 2a confirmed) are in line with skeptical concerns discussed in the media and in public due to the unusually short-term development of the COVID-19 vaccine based on a new vaccination principle (Anand and Stahel, 2021; Krause et al., 2023; Peretti-Watel et al., 2024). In contrast, *Collective Complacency^R^*, *Responsibility*, and *Compliance* regarding readiness for COVID-19 vaccination proved to be particularly high (Hypothesis 2b confirmed). This reflects the urgent public relevance of overcoming health risks resulting from the pandemic, esp. for vulnerable risk groups (Anas et al., 2023), as well as the restrictions in public, social, and economic life (Sprengholz et al., 2021) caused by the comprehensive infection protection measures (Terrell et al., 2023).

Contrary to Hypothesis 2c, the benefits of COVID-19 vaccination seem to subordinate potential constraints associated with vaccination (e.g., immediate vaccination reaction). However, a considerable interaction effect in line with Hypothesis 3 must be regarded: If there is no influenza vaccination recommendation, the *Contraints^R^* differences between the two vaccination types are significantly greater than if influenza vaccination is recommended. Similarly, *Collective Responsibility* also differs for the two types of vaccination only if an influenza vaccination recommendation is given, which is in line with expectations, as these people are more likely to have contact with risk groups, e.g., professionally or due to their own age. No interaction effect with the influenza vaccination recommendation was found for the other 7C scales (Hypothesis 3 rejected), indicating that these responses on vaccination readiness were largely independent of the risk perception for oneself and for social interaction partners.

Geiger et al. (2021) emphasize the research desideratum to examine the differential measurement properties of the 7C subscales in various application contexts and for different types of vaccination. In their study, the psychometric properties of the seven individual scales proved to be rather unsatisfactory, as the cross-scale main factor “General vaccination readiness” turned out to be the dominant source of variance. Our study made a significant contribution to this by demonstrating the strong vaccine type-dependent profiles according to Hypothesis 2. Furthermore, the selective responsiveness of the individual scales (Hypothesis 2b), which are theoretically most closely related to the special circumstances of the COVID-19 pandemic (especially infection control, social coexistence; Sprengholz et al., 2021; Watson et al., 2022), underpin their factorial validity (Messick, 1995). In addition, the satisfactory internal consistencies (*ω* > 0.770) of 6 of the seven individual scales with moderate scale intercorrelations for both vaccination types also indicate an overall psychometrically stable factorial structure. It should be noted here that Geiger et al. (2021) determined the internal consistencies according to McDonald’s *ω* within the framework of bi-factor modeling, where the main factor, “General vaccination readiness” related variance, is partialized out before calculating the factor reliabilities. Thus, the reliability estimates reflect the internal consistency of the residual variance components that are not explained by the main factor. For the *Calculation^R^* factor, however, the internal consistency estimates of *ω* = 0.537 and 0.583 are similar to those of Geiger et al. (2021; *ω* = 0.520): Since only the *Calculation^R^* items, in contrast to the other 18 items, proved to be very weakly associated with the general factor, in our study similar estimates resulted as for the bi-factor modeling (Geiger et al., 2021). Furthermore, in accordance with Geiger et al. (2021), Schindler et al. (2020), and Neufeind et al. (2021), the *Calculation^R^* scale was found to be strikingly weaker associated with the validation criteria in the correlation and regression analyses than the other 7C subscales. The contents of *Calculation^R^* items reflect the assumption that a conscious risk–benefit calculation indicates a limited readiness to be vaccinated. However, the empirical findings suggest that a cost–benefit assessment could also indicate a desirable characteristic of rational user behavior in people who are willing to be vaccinated (Rees et al., 2022; Wilder-Smith et al., 2017).

The findings of Geiger et al. (2021) regarding the criteria validity of the 7C scale were well replicated, with 84.1% of the variance in importance assessment and information modeling for dichotomous vaccination status explained (Nagelkerke-*R*^2^ = 0.695). The lower variance explanation for influenza vaccination (75.8%; Nagelkerke-*R*^2^ = 0.457) may indicate that willingness to be vaccinated against COVID-19 is more closely linked to actual behavior (Betsch et al., 2015), likely due to the heightened emphasis on individual and social protection during the pandemic (Chevallier et al., 2021). This is further supported by the fact that for COVID-19 vaccination, vaccination status is significantly more correlated with COVID-19-related *Constraints^R^* and *Collective Responsibility* (*r* = 0.628, 0.740) than is the case for influenza vaccination status and the corresponding influenza-related 7C subscales (*r* = 0.489, 0.480).

### 4.1 Limitations

One of the limitations of this study was that it used only a convenience sample, which was carried out via online channels under the special contact restrictions of the COVID-19 pandemic. Due to the corresponding representativeness restrictions, conclusions about the general conditions in the German population are limited. However, as the questions are not epidemiological but instead refer in particular to differential measurement properties of the 7C scale for two vaccination types, this restriction was accepted as being of minor importance for the study design. The information on vaccination status and the criteria for an influenza vaccination recommendation are based on self-reporting within an anonymous online collection setting. Although there are no indications that incorrect information may have been provided here (e.g., due to memory biases, social desirability, or self-serving biases), it would have been desirable to objectively verify this information. The sample is dominated by young people with a more academic background. A comparatively high percentage of people were vaccinated against COVID-19. Despite the low average age, it was possible to include 135 people (42.6%) in the study for whom an influenza vaccination recommendation was available. Contrary to Hypothesis 3, the presence of an influenza vaccination recommendation only had a marginal effect on the data structure. The associated distortions should be rather moderate. Despite the multitude of statistical tests calculated, economic data analysis was realized by prioritizing the hypotheses (3 main hypotheses), testing the main hypotheses using an integrated ANOVA and hypothesis-specific *α*-error adjustment, and applying Bonferroni correction of the significance level. Although the high COVID-19 vaccination rate (91%) is highly encouraging for infection protection, it statistically led to the problem of a very skewed distribution of the dichotomous criterion variable in the logistic regression. Accordingly, the ß-weight tests exhibit low test power. Thus, the non-significance (after Bonferroni correction) of all predictors should be interpreted with caution.

The original items of the 7C scale were reformulated specifically for COVID-19 and influenza vaccination. The influenza vaccination-related adaptation proved to be challenging for the *Compliance* scale, as its contents refer to restrictive protective measures (e.g., exclusion of non-vaccinated persons from public events) appearing inappropriately restrictive for influenza vaccination. Due to this adaptation, the content and difficulty of the corresponding COVID-19 vs. influenza-related *Compliance* items may limit the fairness of the comparison between vaccination types. Since individual and public living conditions were subject to considerable changes during and after the pandemic due to the dynamics of the risk of infection, the findings presented can only claim validity with regard to the final phase of the pandemic. For this observational study, it should be noted that causal conclusions are not permissible due to the correlative statistics and mean value comparisons. As we have chosen a diagnostic or assessment focus for the study content, this is not considered critical in terms of our study objective. However, no statements about mechanisms of action or time courses may be regarded as confirmed by the study findings. It would have been desirable to be able to analyze qualitative survey data in addition to the quantitative data. This could have contributed to a deeper understanding of the significance of the assessed data, particularly with regard to the qualitative differences between the two forms of vaccination. Such a mixed-methods approach could be considered in future research to test and increase the validity of the study findings on vaccine-specific readiness to be vaccinated.

### 4.2 Future research

The conditions underlying the hypotheses on the different readiness to be vaccinated for the two types of vaccine are subject to changes over time (Abate et al., 2024; Harada and Watanabe, 2021; Leonardelli et al., 2023): By now, the special pandemic-related living conditions have passed, and the risk of COVID-19 infections is no longer a dominating factor in individual and societal everyday life. This is due to both a higher immunity in the population and a weakening of the virus variants (RKI, 2024b). In this respect, the experience of threat should be reduced, and it could be assumed that this will have an impact at least on the facets of *Collective Responsibility*, *Complacency^R^*, *Constraints^R^* and *Compliance* for COVID-19.

In contrast, the increasing experience and approval of COVID-19 vaccinations (Geoghegan et al., 2020) should lead to an improvement in the previously lower *Confidence* and *Conspiracy^R^*. Thus, it would be instructive to investigate whether and which vaccine- and risk-related aspects (Dombrádi et al., 2021; Enria et al., 2024; Jabkowski et al., 2023; Dubé et al., 2013; SteelFisher et al., 2023) and associated changes in public perception influence attitudes toward COVID-19 vaccination. This should provide valuable insights into the nature of the construct of vaccination readiness and contribute to a differentiated and more valid understanding (Dubé et al., 2013; MacDonald, 2015; Spier, 2001). A future convergence of readiness for vaccination against COVID-19 and influenza would indicate that more general, vaccine-independent attitudes determine vaccination readiness and its structural components.

Across different countries, varying levels of trust in government, healthcare systems, and social influences were found to be determinants of vaccine acceptance (Abate et al., 2024; Alper et al., 2020; Shi et al., 2024; Huang et al., 2022; Leong et al., 2022; Rosen et al., 2021). Future studies should strive to shed light on how different cultural contexts interact with individual vaccination-related beliefs and attitudes (Petráš et al., 2022; Shi et al., 2024). First, for a deeper understanding of vaccination-related attitudes and valid conception interventions, the level of the individual and the level of informing and supplying health authorities must be understood in their interplay. For e.g., trust in public health authorities and the government is known to moderate vaccine acceptance systematically (Leong et al., 2022). Considering the sub-factors of vaccination readiness according to the 7C approach enables a more differentiated understanding (i) of the facets of the construct of vaccination readiness as well as (ii) the influences of the healthcare system and the provision of information on these construct facets (Guan et al., 2024; Terrell et al., 2023). Evidence on these aspects is important in order to shape communication and political measures in such a way that the acceptance of the vaccine can be effectively increased. Second, research should investigate the impact of social influence and conformity in shaping vaccination attitudes and behavior, particularly in contrast to collectivist cultures like China, where community norms affect individual choices more strongly, and more individualistic Western cultures (Shi et al., 2024). Finally, further work is needed on developing and testing multi-layered interventions, such as combining education, regulatory frameworks, and behavioral nudges, to ensure that strategies are effective across varying health systems and socio-political contexts (Abate et al., 2024; Petráš et al., 2022; Shi et al., 2024; Terrell et al., 2023).

It is reasonable to assume that the fit between the aspects of public health intervention and individual, vaccine-specific attitudes is crucial to the success of interventions to increase willingness to vaccinate (Terrell et al., 2023). The study findings presented here are exemplary in helping to provide a differentiated picture of vaccination readiness in contrast between the two types of vaccination. If future research would succeed in investigating temporal trends in different countries or depending on different intervention approaches, this should prove to be particularly informative for studying the construct of readiness to vaccinate, both with regard to the characteristics of the construct itself and with regard to the determinants that affect its characteristics.

Finally, the fundamental importance of a differentiated understanding of vaccination readiness for appropriate communication on the subject of vaccination, especially between healthcare professionals and laypeople, needs to be emphasized (Ulrich et al., 2022). The COVID-19 pandemic has shown impressively the importance of vaccinations as a crucial tool for preventing severe illnesses, hospitalization, and death from infectious diseases (Wu et al., 2024; Du et al., 2024). Vaccination readiness is a central determinant of the successful implementation and utilization of vaccinations in prevention practice (Geiger et al., 2021; Harada and Watanabe, 2021). Thus, health professionals need to be sensitized and trained to understand and address people’s vaccination-related attitudes effectively (Chou et al., 2021; Dubé et al., 2013; European Centre for Disease Prevention and Control (ECDC), 2024; MacDonald, 2015; WHO, 2021a,b).

The results presented here clarify critical vaccine-specific attitudinal profiles, helping professionals to adopt a more competent and informed approach to vaccination attitudes (Jackson et al., 2023). In a comparison of influenza and COVID-19 vaccination, it was shown that it is important to distinguish sub-facets of vaccination readiness in order to understand and validly assess the individual’s perspective and attitudes toward vaccinations. From the 7C perspective, the overarching construct *Vaccination Readiness* is differentiated into attitudes toward the vaccine or vaccination offer (*Confidence*), perception of threat and the need for vaccination (*Complacency^R^*), cost–benefit assessment (*Calculation^R^*), efforts required to receive the vaccine (*Constraints^R^*), willingness to protect the health of other people and to contribute to community protection (herd immunity; *Collective Responsibility*), adherence to recommended and socially agreed behaviors (*Compliance*), as well as resistance to *Conspiracy^R^* (esp. fake news and myths). Depending on the individual, medical, and social situation, it is necessary to reflect on these sub-facets to adequately address the phenomenon of vaccination readiness or vaccination hesitancy both situationally and generally (Abate et al., 2024; Chou et al., 2021; Ulrich et al., 2022; WHO, 2021a,b). Depending on the specific risks of infection and the threat of infectious diseases, these findings can help health professionals support those receiving care in making informed decisions about the prevention of infectious diseases through vaccination.

## Data Availability

The raw data supporting the conclusions of this article will be made available by the authors, without undue reservation.

## Footnotes

^1^www.dgps.de/serviceangebote/ethikkommission/; registration number MAW 112018.
