# Supplementary material for: Readiness for influenza and COVID-19 vaccination in Germany: a comparative analysis
Source: Front Psychol. 2024 Oct 17;15:1437942. doi: 10.3389/fpsyg.2024.1437942 (PMC11528425; doi:10.3389/fpsyg.2024.1437942)
Supplement: Supplementary file 1 [file Table_1.DOCX]

**Supplemental material 1:** Adapted and original formulation of thr 21 items of the 7C scale.

| **Adapted item formulations for influenza vaccination** | **Adapted item formulations for COVID-19 vaccination** | **Original items oft he 7C scale (Geiger et al., 2021)** |
| --- | --- | --- |
| **Confidence** | | |
| Nebenwirkungen treten nach der Grippeschutzimpfung selten auf und sind für mich nicht schwerwiegend.  [Side effects rarely occur after the flu vaccination and are not severe for me.] | Nebenwirkungen treten nach der COVID-19-Impfung selten auf und sind für mich nicht schwerwiegend.  [Side effects rarely occur after the flu vaccination and are not severe for me.] | Vaccination side effects occur rarely and are not severe for me. |
| Politische Entscheidungen zur Grippeschutzimpfung sind wissenschaftlich fundiert.  [Political decisions about flu vaccinations are scientifically grounded.] | Politische Entscheidungen zur COVID-19-Impfung sind wissenschaftlich fundiert.  [Political decisions about COVID-19 vaccinations are scientifically grounded.] | Political decisions about vaccinations are scientifically grounded. |
| Ich bin überzeugt davon, dass die zuständigen Behörden nur wirksame und sichere Impfstoffe gegen Grippe zulassen.  [I am convinced the appropriate authorities do only allow effective and safe flu vaccines.] | Ich bin überzeugt davon, dass die zuständigen Behörden nur wirksame und sichere Impfstoffe gegen COVID-19 zulassen.  [I am convinced the appropriate authorities do only allow effective and safe COVID-19 vaccines.] | I am convinced the appropriate authorities do only allow effective and safe vaccines. |
| **Complacency^R^** | | |
| Ich brauche keine Grippeschutzimpfung, weil Infektionskrankheiten bei mir nicht schlimm verlaufen.  [I do not need flu vaccination because infectious diseases do not hit me hard.] | Ich brauche keine COVID-19-Impfung, weil Infektionskrankheiten bei mir nicht schlimm verlaufen.  [I do not need COVID-19 vaccination because infectious diseases do not hit me hard.] | I do not need vaccinations because infectious diseases do not hit me hard. |
| Die Grippeschutzimpfung ist für mich überflüssig, weil ich sowieso selten krank werde.  [Flu vaccinations is unnecessary for me because I rarely get ill anyway.] | Die COVID-19-Impfung ist für mich überflüssig, weil ich sowieso selten krank werde.  [COVID-19 vaccination is unnecessary for me because I rarely get ill anyway.] | Vaccinations are unnecessary for me because I rarely get ill anyway. |
| Ich lasse mich impfen, weil es zu riskant ist, Grippe zu kriegen.  [I get vaccinated because it is too risky to get the flu.] | Ich lasse mich impfen, weil es zu riskant ist, COVID-19 zu kriegen.  [I get vaccinated because it is too risky to get the COVID-19.] | I get vaccinated because it is too risky to get infected. |
| **Constraints^R^** | | |
| Ich kümmere mich darum, die Grippeschutzimpfung rechtzeitig zu erhalten.  [I make sure to receive the flu vaccination in good time.] | Ich kümmere mich darum, die COVID-19-Impfung/Auffrischimpfungen rechtzeitig zu erhalten.  [I make sure that to receive the COVID-19 vaccination/booster vaccinations in good time.] | I make sure to receive the most important vaccinations in good time. |
| Die Grippeschutzimpfung ist mir so wichtig, dass ich andere Sachen zurückstelle, um mich impfen zu lassen.  [Flu vaccination is so important to me that I prioritize getting vaccinated over other things.] | Die COVID-19-Impfungen sind mir so wichtig, dass ich andere Sachen zurückstelle, um mich impfen zu lassen.  [COVID-19 vaccination is so important to me that I prioritize getting vaccinated over other things.] | Vaccinations are so important to me that I prioritize getting vaccinated over other things. |
| Ich nehme die Grippeschutzimpfung nicht in Anspruch, weil Impfen lästig ist.  [I miss out flu vaccination(s) because vaccination is bothersome.] | Ich nehme die COVID-19-Impfung(en) nicht in Anspruch, weil Impfen lästig ist.  [I miss out COVID-19 vaccination(s) because vaccination is bothersome.] | I sometimes miss out on vaccinations because vaccination is bothersome. |
| **Calculation^R^** | | |
| Ich lasse mich gegen Grippe impfen, wenn ich für mich keine Nachteile sehe.  [I get vaccinated against flu when I do not see disadvantages for me.] | Ich lasse mich gegen COVID-19 impfen, wenn ich für mich keine Nachteile sehe.  [I get vaccinated against COVID-19 when I do not see disadvantages for me.] | I get vaccinated when I do not see disadvantages for me. |
| Ich lasse mich nur dann gegen Grippe impfen, wenn die Vorteile deutlich die Risiken überwiegen.  [I only get vaccinated against flu when the benefits clearly outweigh the risks.] | Ich lasse mich nur dann gegen COVID-19 impfen, wenn die Vorteile deutlich die Risiken überwiegen.  [I only get vaccinated against COVID-19 when the benefits clearly outweigh the risks.] | I only get vaccinated when the benefits clearly outweigh the risks. |
| Ich überlege jedes Jahr neu, ob ich die Grippeschutzimpfung brauche.  [I consider whether I need the flu vaccination every year.] | Ich überlege bei jeder COVID-19-Impfung genau, ob ich sie brauche.  [For each COVID-19 vaccine, I carefully consider whether I need it.] | For each vaccine, I carefully consider whether I need it. |
| **Collective Responsibility** | | |
| Ich lasse mich auch gegen Grippe impfen, weil für mich der Schutz gefährdeter Risikogruppen wichtig ist.  [I also get vaccinated aganist flu, because protecting vulnerable risk groups is important to me.] | Ich lasse mich auch gegen COVID-19 impfen, weil für mich der Schutz gefährdeter Risikogruppen wichtig ist.  [I also get vaccinated aganist COVID-19, because protecting vulnerable risk groups is important to me.] | I also get vaccinated because protecting vulnerable risk groups is important to me. |
| Ich sehe das Impfen als eine gemeinschaftliche Aufgabe gegen die Verbreitung von Grippe.  [I see vaccination as a collective task against the spread of flu.] | Ich sehe Impfen als eine gemeinschaftliche Aufgabe gegen die Verbreitung von COVID-19.  [I see vaccination as a collective task against the spread of COVID-19.] | I see vaccination as a collective task against the spread of diseases. |
| Ich lasse mich auch deswegen gegen Grippe impfen, weil ich dadurch andere Menschen schütze.  [I also get vaccinated against flu because I am thereby protecting other people.] | Ich lasse mich auch deswegen gegen COVID-19 impfen, weil ich dadurch andere Menschen schütze.  [I also get vaccinated against COVID-19 because I am thereby protecting other people.] | I also get vaccinated because I am thereby protecting other people. |
| **Compliance** | | |
| Personen ohne Grippeschutzimpfung sollten während der Grippesaison in besonderem Maße auf die Hygieneregeln achten.  [People without a flu vaccination should pay particular attention to hygiene rules during the flu season.] | Es sollte möglich sein, Menschen von öffentlichen Veranstaltungen (z.B. Konzerten) auszuschließen, wenn sie nicht gegen COVID-19 geimpft sind.  [It should be possible to exclude people from public events (e.g. concerts) if they have not been vaccinated against COVID-19.] | It should be possible to exclude people from public activities (e.g., concerts) when they are not vaccinated against a specific disease. |
| Es sollte spezielle Regeln für Personen geben, die sich trotz Grippeschutzimpfempfehlung der Gesundheitsbehörden nicht impfen lassen wollen.  [There should be special rules for people who do not get vaccinated against flu despite the health authorities' recommendation to do so.] | Es sollte Konsequenzen für Personen geben, die nicht den COVID-19-Impfempfehlungen der Gesundheitsbehörden folgen.  [It should be possible to sanction people who do not follow the COVID-19-vaccination recommendations by health authorities. | It should be possible to sanction people who do not follow the vaccination recommendations by health authorities. |
| Die Gesundheitsbehörden sollten alle nötigen Mittel einsetzen, um hohe Grippeschutz-Impfraten zu erreichen.  [The health authorities should use all possible means to achieve high flu vaccination rates.] | Die Gesundheitsbehörden sollten alle nötigen Mittel einsetzen, um hohe COVID-19-Impfraten zu erreichen.  [The health authorities should use all possible means to achieve high COVID-19 vaccination rates.] | The health authorities should use all possible means to achieve high vaccination rates. |
| **Conspiracy^R^** | | |
| Die Grippeschutzimpfung verursacht Erkrankungen und Allergien, die schlimmer sind als die Krankheit, gegen die sie schützen soll.  [The flu vaccination causes diseases and allergies that are more serious than the diseases they ought to protect from.] | Die COVID-19-Impfung verursacht Erkrankungen und Allergien, die schlimmer sind als die Krankheit, gegen die sie schützen soll.  [The COVID-19 vaccination causes diseases and allergies that are more serious than the diseases they ought to protect from.] | Vaccinations cause diseases and allergies that are more serious than the diseases they ought to protect from. |
| Die Gesundheitsbehörden beugen sich bezüglich der Grippeschutzimpfung blind der Macht und dem Einfluss der Pharmakonzerne.  [Health authorities knuckle under to the power and influence of pharmaceutical companies regarding flu vaccination.] | Die Gesundheitsbehörden beugen sich bezüglich der COVID-19-Impfung blind der Macht und dem Einfluss der Pharmakonzerne.  [Health authorities knuckle under to the power and influence of pharmaceutical companies regarding COVID-19 vaccination.] | Health authorities knuckle under to the power and influence of pharmaceutical companies. |
| Die Grippeschutzimpfung enthält Chemikalien in giftigen Dosierungen.  [Flu vaccinations contain chemicals in toxic doses.] | Die COVID-19-Impfung enthält Chemikalien in giftigen Dosierungen.  [COVID-19 vaccinations contain chemicals in toxic doses.] | Vaccinations contain chemicals in toxic doses. |
