# Supplementary material for: Readiness for influenza and COVID-19 vaccination in Germany: a comparative analysis
Source: Front Psychol. 2024 Oct 17;15:1437942. doi: 10.3389/fpsyg.2024.1437942 (PMC11528425; doi:10.3389/fpsyg.2024.1437942)
Supplement: Supplementary file 2 [file Table_2.docx]

**Supplemental material 2:** Sample characteristics (N = 317)

| **Age**  [Min; 1st Q^a)^; Med; 3rd Q; Max] | M = 29.95 (SD = 16.12)  [18; 21; 23; 29; 85] |
| --- | --- |
| **Socio-economic state (MacArthur scale)**  [Min; 1st Q; Med; 3rd Q; Max] | M = 6.22 (SD = 1.53)  [2; 5; 5; 7; 10] |
| **Gender** | **N (%)** |
| female | 234 (73.8%) |
| male | 83 (26.2%) |
| **Family status** |  |
| Married | 49 (15.4%) |
| Divorced / Widowed | 8 (2.2%) |
| Single, steady partner | 125 (39.4%) |
| Single, without partner | 135 (42.6%) |
| **High school graduation** (Abitur) | 287 (90.6%) |
| **Mother tongue**: German | 300 (94.6%) |
| **Vaccination** |  |
| COVID-19 – not complete | 17 (5.4%) |
| COVID-19 – cmplete | 269 (84.9%) |
| Influenza – at least once | 174 (54.9%) |
| Common vaccinations (e.g. tetanus, polio) | 307 (96.8%) |
| **Influenza vaccination recommendation** | 135 (42.6%) |
| Contact person of a person at risk | 71 (22.4%) |
| Increased job-related risk | 51 (16.1%) |
| Older age (from 60 years) | 32 (10.1%) |
| Increased individual health risk | 31 (9.8%) |
| Resident of retirement/nursing home | 7 (2.2%) |
| Pregnant | 2 (0.6%) |

^a)^ Q = Quartile
